# Equivalent values between anterior vertebral height, wedge ratio, and wedge angle for evaluating vertebral mobility and deformity in osteoporotic vertebral fractures: a conventional observational study

**DOI:** 10.1186/s13018-023-03758-w

**Published:** 2023-04-08

**Authors:** Kozo Sato, Masakazu Kogawa, Yuichiro Yamada, Masahiro Yamashiro, Fumio Kasama, Michimasa Matsuda

**Affiliations:** 1 Department of Orthopaedic Surgery, Matsuda Hospital, 17-1 Sanezawa Aza Tatsutayashiki, Izumi-Ku, Sendai, Miyagi 981-3217 Japan; 2 Centre for Orthopaedic and Trauma Research, Discipline of Orthopaedics and Trauma, The University of Adelaide, North Terrace and George St., Adelaide, SA 5005 Australia

**Keywords:** Anterior vertebral height, Wedge ratio, Wedge angle, Vertebral mobility, Vertebral deformity, Equivalent values, Osteoporotic vertebral fracture

## Abstract

**Background:**

Vertebral mobility (V-mobility) has been used to diagnose fresh osteoporotic vertebral fractures (OVFs) and determine bone union by setting cutoff values for these purposes. V-mobility is the difference in vertebral height on dynamic radiographs taken in the sitting and lateral decubitus or supine positions. The dimensions for V-mobility were presented as anterior vertebral height (Ha; mm), wedge ratio (WR; %), and wedge angle (WA; °) in previous reports. This study was performed to obtain WR and WA values equivalent to V-mobility of 1.0 mm in Ha.

**Methods:**

Lateral radiographs of 284 OVFs (grade 1–3 deformed vertebrae) from T11 to L2 were obtained from 77 patients with OVF. V-mobility presented as Ha, posterior vertebral height, and WA was obtained by the difference in these dimensions on dynamic radiographs. The WR and WA values equivalent to 1.0 mm in Ha were obtained by dividing the V-mobility values for WR and WA by that for Ha.

**Results:**

The mean WR values corresponding to 1.0 mm in Ha for grade 1, 2, and 3 vertebrae were 3.2% ± 1.4%, 3.2% ± 0.9%, and 3.4% ± 1.0%, respectively, and the corresponding value for grade 1–3 vertebrae was 3.3% ± 1.0%. The mean WA values corresponding to 1.0 mm in Ha for grade 1, 2, and 3 vertebrae were 1.5° ± 0.8°, 1.5° ± 0.6°, and 1.5° ± 0.8°, respectively, and the corresponding value for grade 1–3 vertebrae was 1.5° ± 0.7°.

**Conclusions:**

The WR and WA values equivalent to V-mobility of 1.0 mm in Ha were 3.3% and 1.5°, respectively, in grade 1–3 vertebrae. These findings may be useful to secure a reliable value of V-mobility of OVFs using simultaneous measurements in three dimensions (Ha, WR, and WA) in clinical practice and to establish cutoff values for V-mobility to determine bone union.

## Background

Osteoporotic vertebral fractures (OVFs) commonly occur in older adults, particularly women [1], and the population with OVFs in Japan is predicted to further increase until 2030–2035 [2]. OVFs are usually treated conservatively with good outcomes [3]. Conservative treatments for OVFs include bed rest, analgesic medication, physiotherapy, bracing [4], and exercises to recover muscle atrophy arising from disuse during the initial period of bed rest [5].

Bed rest during the early stage of OVF treatment is reportedly very effective for achieving a high healing rate and avoiding delayed onset of neurological deficits [6–9]. Orthoses are frequently used to relieve fracture-related back pain [10], prevent vertebral collapse, and promote bone healing. However, the efficacy of wearing an orthosis has not been established [10–13]. The efficacy and limitations of conservative treatment modalities should be clarified through further clinical trials by objective evaluation of the OVF status, particularly by setting a cutoff value to determine bone union.

Vertebral mobility (V-mobility) is a very useful parameter for objective evaluation of the OVF status [14] and has been used to diagnose fresh OVFs [15, 16], follow-up OVFs [9], determine bone union [7, 9, 17–21], and predict bone union at an early OVF stage [9, 22]. V-mobility is defined as the difference in vertebral height on dynamic lateral radiographs taken in weight-bearing (standing or sitting [SIT]) and non-weight-bearing (lateral decubitus [DEC] or supine [SUP]) positions, and it can be numerically presented as anterior vertebral height (Ha; mm), posterior vertebral height (Hp; mm), wedge ratio (WR; %), and wedge angle (WA; °).

Only a few previous reports have described cutoff values for V-mobility to detect bone union in OVFs, namely Ha of ≤ 1.0 mm [9, 18], Ha of ≤ 2.0 mm [21], WR of ≤ 5% [17], and WA of ≤ 5° [7, 19, 20] on dynamic radiographs. These cutoff values were likely set based on their own concepts and have been presented as different dimensions (Ha, WR, or WA). Therefore, it is necessary to determine equivalent values between Ha, WR, and WA (i.e., WR and WA values equivalent to 1.0 mm in Ha) to compare the results evaluated by these different parameters.

In our previous study [23], we clarified WR and WA values equivalent to 1.0 mm in Ha for the first time based on morphometric values measured on lateral radiographs of normal vertebrae and stable previous OVFs from T11 to L2, as described in more detail in the Discussion section. The mean WR value equivalent to 1.0 mm in Ha (WR/Ha) and the corresponding WA value (WA/Ha) were found to be 3.5% and 1.5°, respectively, for vertebrae with grade 1–3 deformities.

The present study was performed to obtain WR and WA values equivalent to 1.0 mm in Ha by calculating V-mobility values for WR and WA corresponding to V-mobility of 1.0 mm in Ha using radiographs of mobile clinical OVFs under treatment at daily clinical practices. We compared these values with our previous results to determine the reliability of the results of these two studies.

## Materials and methods

The present study was approved by the Committee of Medical Ethics of Matsuda Hospital (June 7, 2022; approval number, 4–1). Written informed consent was obtained from all patients included in the study. The study was a conventional observational study.

### Patients

Lateral dynamic radiographs of the thoracolumbar vertebrae from T11 to L2 were obtained from patients with OVF treated as inpatients in the convalescent rehabilitation ward in our hospital from October 2009 to March 2020. Lateral radiographs showing the following characteristics were excluded from the study: concave vertebrae with depressed endplates only, severely collapsed burst fractures, vertebrae with injured margins of vertebral bodies that could not be detected to set measurement points, and vertebrae with elliptic endplates caused by inappropriate centering of the X-ray beam on the injured vertebrae. Vertebrae with pathological fractures (tumor or infection) were also excluded. In total, 426 lateral dynamic radiographs of OVFs from T11 to L2 were obtained from 77 patients with OVF (62 women, 15 men; mean age, 81 years; age range, 55–98 years) with a body height of 149.5 ± 7.0 cm and body mass index of 22.0 ± 3.5 kg/m^2^. The mean number of radiographs that were taken during follow-up and available for this study was 5.5 radiographs per patient.

### Radiographs and morphometric assessment

**Fig. 1:**
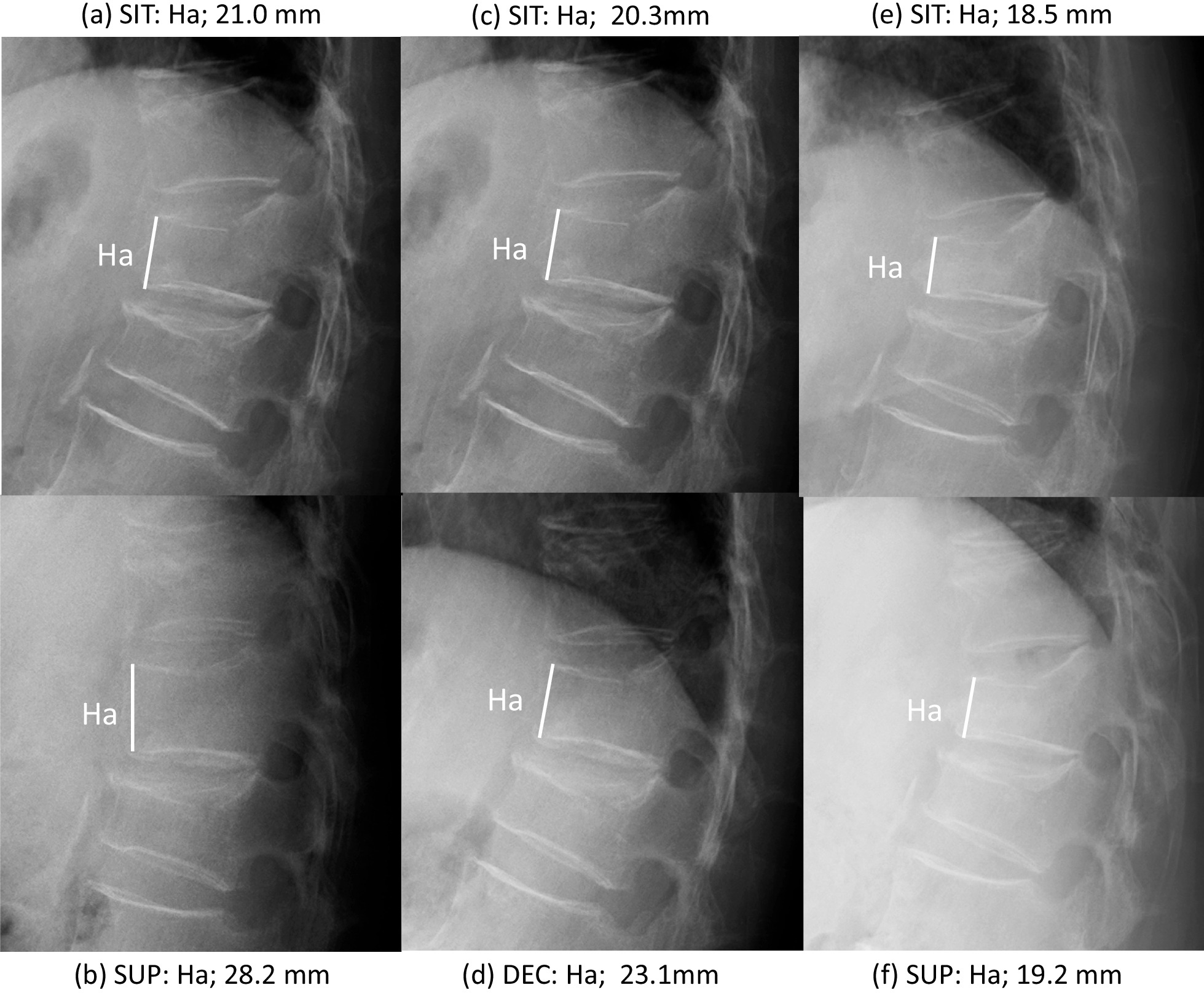
Radiological evaluation of one patient with a fresh OVF at T12 and a stable previous OVF at L1. (**a**, **b**) Lateral radiographs obtained in SIT and SUP to evaluate V-mobility in the initial evaluation at 13 days after OVF onset. (**c**, **d**) Radiographs obtained during treatment in SIT and DEC to show V-mobility at 61 days after OVF onset. (**e**, **f**) Radiographs obtained in SIT and SUP in the final evaluation to detect radiological bone union at 146 days after OVF onset. *OVF* osteoporotic vertebral fracture, *SIT* sitting position, *SUP* supine position, *DEC* lateral decubitus position, *V-mobility* vertebral mobility

We used two radiographic techniques: radiofluorescence radiography with a fixed tube-to-film distance of 120 cm and computed radiography. The images were obtained with a RadiForce MX215 (EIZO Corporation, Hakusan, Ishikawa, Japan) and had a resolution of 1200 × 1600 pixels. The enlargement difference in radiographs taken with radiofluorescence radiography and computed radiography was adjusted by 3% because of the different table-to-film distance (2.1 cm) [23], and the enlargement differences in the SIT and DEC or SUP radiographs were adjusted with reference to the Ha values of adjacent intact vertebrae. The initial evaluation was principally based on SIT and SUP radiographs to demonstrate the V-mobility at baseline. Subsequent radiographs for follow-up were taken in SIT and DEC to prevent loosening of the fracture site in SUP, and radiographs to ascertain bone union were taken in SIT and SUP (Fig. 1).

**Fig. 2:**
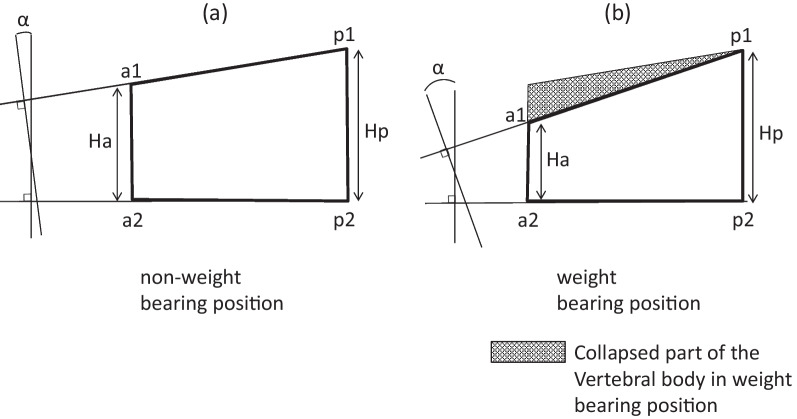
The four measurement points on the vertebral images were set at the anterior superior (a1), anterior inferior (a2), posterior superior (p1), and posterior inferior (p2) margins of the vertebral body. Ha and Hp vertebral dimensions were defined as the distances from a1 to a2 and from p1 to p2, respectively. WA was defined as the angle between two lines through a1 and p1 and through a2 and p2. WR (%) was calculated as Ha / Hp × 100 (%). Lateral radiographs of an osteoporotic vertebral fracture are shown in the (**a**) non-weight-bearing position and (**b**) weight-bearing position. The shadowed area indicates the collapsed part of the vertebral body in the weight-bearing position (V-mobility). *Ha* anterior vertebral height, *Hp* posterior vertebral height, *WR* wedge ratio, *WA* wedge angle, *V-mobility* vertebral mobility

The measurement points were set at the four corners of the vertebral body on lateral radiographs, and Ha (mm), Hp (mm), and WA (°) were measured (Fig. 2). WR (%) was calculated as Ha/Hp × 100 (%). The V-mobility values for WR and WA equivalent to 1.0 mm of Ha were obtained by dividing the V-mobility values for WR and WA by that for Ha, giving WR/Ha and WA/Ha, respectively.

Precision errors for measurements were calculated for Ha, Hp, and WA on the radiographs of 20 randomly selected OVFs and expressed as percentage coefficients of variation (CVs) as follows. Each dimension was measured five times on five differently enlarged images for each of the 20 OVFs, and the CV was calculated for each OVF. The mean CV values for the 20 OVFs were obtained with standard deviations (SDs). Reliabilities of vertebral dimension measurements were assessed as follows. The dimensions of Ha, Hp, and WA were measured on the radiographs of 30 randomly selected OVFs by one author (K.S.). The intraobserver reliabilities for the measurements of these three dimensions were assessed by intraclass correlation coefficients based on three sets of measurements with an interval of > 1 week.

### Grading of V-deformity

The extent of V-deformity was presented as WR and graded from 1 to 3 with reference to the grading system established by Genant et al. [24]: grade 1, mildly deformed (WR of 75% to < 80%); grade 2, moderately deformed (WR of 60% to < 75%); and grade 3, severely deformed (WR of < 60%).

### Statistical analysis

The differences in the mean morphometric and V-mobility values between the grade 1–3 vertebrae were analyzed by one-way analysis of variance followed by Bonferroni’s post hoc test. The differences in WR/Ha and WA/Ha at each level of the spine (T11–L2) were analyzed using Bonferroni’s test. The mean WR/Ha and WA/Ha values between L1 and L2 were analyzed using Student’s *t* test because there were no grade 1 vertebrae at T11 and T12 in this study. Data are presented as mean ± SD. Statistical analysis results were considered significant at *p* < 0.05. All analyses were performed using BellCurve for Excel (version 3.21) (Social Survey Research Information, Tokyo, Japan).

## Results

The 426 OVFs were categorized into radiologically stable and unstable OVFs according to the results of the V-mobility measurements. We then excluded the 142 radiologically stable OVFs, comprising bone union (V-mobility of ≤ 1.0 mm in Ha on SIT and SUP radiographs) and semi-union (V-mobility of ≤ 1.0 mm in Ha on SIT and DEC radiographs) without an intravertebral cleft [9], which became stable after treatment. The 284 remaining radiologically unstable OVFs with V-mobility of > 1.0 mm in Ha, defined as non-united OVFs under treatment, were included in the study to calculate the WR and WA values equivalent to 1.0 mm in Ha.

### Morphometric values of Ha, Hp, WR, and WA

Regarding the precision errors for the morphometric values, the percentage CVs for OVFs were 2.9% ± 1.7% for Ha, 2.3% ± 0.7% for Hp, and 4.6% ± 2.2% for WA. The intraobserver reliabilities of the measurements assessed by intraclass correlation coefficients were 0.980 (95% confidence interval [CI]: 0.964–0.990, *p* < 0.001) for Ha, 0.954 (95% CI: 0.917–0.976, *p* < 0. 001) for Hp, and 0.878 (95% CI: 0.791–0.935, *p* < 0.001) for WA.

The mean Ha, Hp, WR, and WA values on SIT and DEC or SUP radiographs for OVFs are shown in Table 1. The mean Ha and WR values decreased and the mean WA values increased as anticipated with advancement from grade 1 to grade 3 vertebrae with a significant difference between each grade of V-deformity. The mean Hp values were only apparently decreased in grade 3 vertebrae. The differences in the mean Ha, Hp, WR, and WA values between each grade of V-deformity on DEC or SUP radiographs were similar to those on SIT radiographs.

**Table 1:** Morphometric values of Ha, Hp, WR, and WA on weight-bearing (SIT) and non-weight-bearing (DEC or SUP) radiographs

Grade	Level	*n*	SIT				DEC or SUP			
			Ha (mm)	Hp (mm)	WR (%)	WA (°)	Ha (mm)	Hp (mm)	WR (%)	WA (°)
Grade 1	L1	4	23.8 ± 1.4	30.9 ± 1.7	77.1 ± 1.7	12.0 ± 1.8	27.5 ± 1.7	31.9 ± 3.3	86.5 ± 6.4	7.1 ± 3.3
	L2	3	21.2 ± 2.3	27.7 ± 3.4	76.6 ± 1.2	10.6 ± 1.6	23.0 ± 3.0	27.2 ± 2.9	84.7 ± 4.3	6.9 ± 2.7
	L1-2	7	22.7 ± 2.2	29.5 ± 2.9	76.9 ± 1.4	11.4 ± 1.7	25.6 ± 3.2	29.9 ± 3.8	85.7 ± 5.2	7.0 ± 2.8
Grade 2	T11	12	16.4 ± 2.3	26.2 ± 3.3	62.5 ± 3.2	16.8 ± 2.1	20.3 ± 3.5	27.2 ± 3.4	74.8 ± 6.7	11.5 ± 2.8
	T12	31	19.8 ± 2.1	30.1 ± 2.1	65.9 ± 4.3	16.8 ± 2.6	23.1 ± 3.0	30.4 ± 2.2	76.1 ± 7.5	12.0 ± 3.9
	L1	37	20.2 ± 2.0	30.8 ± 2.3	65.5 ± 3.8	16.9 ± 2.5	23.2 ± 2.2	30.9 ± 2.3	75.2 ± 7.1	12.6 ± 3.9
	L2	14	21.2 ± 2.0	32.2 ± 3.9	66.1 ± 5.8	17.0 ± 4.6	25.0 ± 2.3	32.3 ± 4.2	78.0 ± 7.3	10.9 ± 4.6
	T11-L2	94	19.7 ± 2.4 ^a^	30.2 ± 3.1	65.3 ± 4.3 ^b^	16.9 ± 2.8 ^b^	23.1 ± 2.9	30.5 ± 3.1	75.9 ± 7.2 ^a^	12.0 ± 3.9 ^a^
Grade 3	T11	18	12.1 ± 3.3	24.2 ± 3.4	49.6 ± 11.4	22.9 ± 6.0	15.6 ± 4.5	24.9 ± 3.7	62.6 ± 15.6	17.0 ± 7.6
	T12	57	13.6 ± 3.6	28.3 ± 3.7	47.8 ± 10.4	24.4 ± 4.3	16.8 ± 3.6	28.5 ± 3.2	58.8 ± 9.9	19.7 ± 4.5
	L1	96	13.3 ± 3.0	28.6 ± 2.7	46.3 ± 8.5	24.6 ± 3.7	16.8 ± 3.9	29.0 ± 2.4	57.9 ± 11.7	19.7 ± 5.4
	L2	12	15.3 ± 3.3	31.3 ± 4.1	48.8 ± 7.5	22.9 ± 1.8	18.4 ± 3.8	31.5 ± 3.6	58.6 ± 10.1	18.9 ± 4.0
	T11-L2	183	13.4 ± 3.3^b,α^	28.3 ± 3.5^α^	47.3 ± 9.4^b,α^	24.2 ± 4.1^b,α^	16.8 ± 3.9^b,α^	28.6 ± 3.2 ^α^	58.7 ± 11.5^b,α^	19.4 ± 5.3^b,α^
Grades 1–3	T11-L2	284	15.7 ± 4.3	28.9 ± 3.5	54.0 ± 12.2	21.5 ± 5.3	19.1 ± 4.7	29.2 ± 3.3	65.0 ± 13.3	16.6 ± 6.1

The differences in the mean morphometric values between each grade of V-deformity were analyzed by one-way analysis of variance followed by Bonferroni’s post hoc test. The grade 1 vertebrae were located at L1 and L2

Grade, grade of vertebral deformity; Ha, anterior vertebral height; Hp, posterior vertebral height; V-deformity, vertebral deformity; WR, wedge ratio; WA, wedge angle; SIT, sitting position; DEC, lateral decubitus position; SUP, supine position

^a^*p* < 0.05, ^b^ *p* < 0.001, grade 1 vs. grade 2 or 3

^α^*p* < 0.001, grade 2 vs. grade 3

### V-mobility presented as Ha, Hp, WR, and WA

The mean V-mobility values presented as Ha, Hp, WR, and WA did not differ significantly between each grade of V-deformity. The mean V-mobility values from T11 to L2 in grade 1–3 vertebrae (*n* = 284) were 3.4 ± 2.1 mm in Ha, 0.3 ± 1.2 mm in Hp, 11.1% ± 7.1% in WR, and 4.8° ± 3.4° in WA (Table 2).

**Table 2:** V-mobility presented as Ha, Hp, WR, and WA, and WR and WA equivalent to V-mobility of 1.0 mm in Ha

Grade	Level	*n*	V-mobility					
			Ha (mm)	Hp (mm)	WR (%)	WA (°)	WR/Ha (%)	WA/Ha (°)
Grade 1	L1	4	3.7 ± 1.4	1.0 ± 1.9	9.4 ± 7.8	5.0 ± 4.1	2.4 ± 1.5	1.2 ± 0.8
	L2	3	1.9 ± 0.9	− 0.5 ± 0.6	8.1 ± 4.8	3.7 ± 2.9	4.2 ± 0.4	1.9 ± 0.8
	L1-2	7	2.9 ± 1.5	0.4 ± 1.6	8.8 ± 6.2	4.4 ± 3.4	3.2 ± 1.4	1.5 ± 0.8
Grade 2	T11	12	3.9 ± 2.1	1.0 ± 1.3	12.2 ± 5.6	5.3 ± 2.7	3.3 ± 0.9	1.5 ± 0.5
	T12	31	3.3 ± 1.9	0.3 ± 1.1	10.2 ± 5.7	4.8 ± 2.9	3.2 ± 1.1	1.5 ± 0.6
	L1	37	3.0 ± 1.6	0.0 ± 0.8	9.7 ± 6.0	4.3 ± 2.6	3.2 ± 0.6	1.5 ± 0.6
	L2	14	3.8 ± 2.2	0.1 ± 1.0	12.0 ± 7.4	6.1 ± 4.5	3.1 ± 1.2	1.5 ± 0.6
	T11-L2	94	3.3 ± 1. 9	0.2 ± 1.0	10.5 ± 6.1	4.9 ± 3.1	3.2 ± 0.9	1.5 ± 0.6
Grade 3	T11	18	3.5 ± 2.8	0.6 ± 1.2	13.0 ± 10.6	5.9 ± 4.3	3.7 ± 1.3	1.8 ± 0.8
	T12	57	3.2 ± 1.8	0.2 ± 1.4	11.0 ± 6.8	4.6 ± 3.1	3.5 ± 0.9	1.5 ± 0.8
	L1	96	3.5 ± 2.5	0.4 ± 1.3	11.6 ± 7.8	4.9 ± 3.7	3.3 ± 1.0	1.4 ± 0.7
	L2	12	3.1 ± 1.6	0.2 ± 1.2	9.7 ± 5.1	4.0 ± 3.2	3.3 ± 0.8	1.4 ± 0.9
	T11-L2	183	3.4 ± 2.3	0.3 ± 1.3	11.4 ± 7.6	4.8 ± 3.5	3.4 ± 1.0	1.5 ± 0.8
Grades 1–3	T11-L2	284	3.4 ± 2.1	0.3 ± 1.2	11.1 ± 7.1	4.8 ± 3.4	3.3 ± 1.0	1.5 ± 0.7

The grade 1 OVFs consisted of those at L1 and L2. The differences in the mean values of V-mobility presented as Ha, Hp, WR, WA, WR/Ha, and WA/Ha between each grade of V-deformity were tested by one-way analysis of variance followed by Bonferroni’s post hoc test. The differences in the mean values of WR/Ha and WA/Ha between each level of the spine at T11–L2 in grade 2 and grade 3 vertebrae were tested by Bonferroni’s test, and those between L1 and L2 in grade 1 were tested by Student’s t test. No significant differences in these tests were found

Grade, grade of vertebral deformity; V-mobility, vertebral mobility; Ha, anterior vertebral height; Hp, posterior vertebral height; WR, wedge ratio; WA, wedge angle; WR/Ha, WR equivalent to V-mobility of 1.0 mm in Ha; WA/Ha, WA equivalent to V-mobility of 1.0 mm in Ha

### WR (%) equivalent to V-mobility of 1.0 mm in Ha: WR/Ha

The mean WR/Ha values from T11 to L2 in each grade of V-deformity were 3.2% ± 1.4%, 3.2% ± 0.9%, and 3.4% ± 1.0%, respectively, with no significant differences (Table 2). The corresponding value for the grade 1–3 vertebrae (*n* = 284) was 3.3% ± 1.0%. The mean WR/Ha values between each level of the spine in each grade of V-deformity did not differ significantly.

### WA (°) equivalent to V-mobility of 1.0 mm in Ha: WA/Ha

The mean WA/Ha values from T11 to L2 in each grade of V-deformity were 1.5° ± 0.8°, 1.5° ± 0.6°, and 1.5° ± 0.8°, respectively, with no significant differences (Table 2). The corresponding value for the grade 1–3 vertebrae (*n* = 284) was 1.5° ± 0.7°. The mean WA/Ha values between each level of the spine in each grade of V-deformity did not differ significantly.

## Discussion

As anticipated, the mean Ha and WR values decreased and the WA value increased with advancement of the V-deformity grade. The mean Hp value apparently decreased in grade 3 vertebrae, indicating the presence of damage to the posterior vertebral wall in OVFs with advanced V-deformity. The mean V-mobility values for WR and WA equivalent to that for 1.0 mm in Ha (WR/Ha and WA/Ha) did not differ significantly between each level of the spine and each grade of V-deformity. The mean WR/Ha and WA/Ha values in grade 1–3 vertebrae in this study were 3.3% and 1.5°, respectively. These values were very similar to the results of our previous study, which demonstrated equivalent values between Ha, WR, and WA for the first time (i.e., 1.0 mm, 3.5%, and 1.5°, respectively) [23].

Several previous reports have described cutoff values for V-mobility to detect bone union, namely Ha of ≤ 1.0 mm [9, 18], Ha of ≤ 2.0 mm [21], WR of ≤ 5.0% [17], and WA of ≤ 5.0° [7, 19, 20] on dynamic radiographs. These previously reported cutoff values for detection of bone union were likely set based on their own concepts or with reference to cutoff values to diagnose acute OVFs. Therefore, we referred to two reports of the cutoff values for diagnosis of acute OVFs. Kawasaki et al. [15] reported that the cutoff value for V-mobility to diagnose fresh OVFs was 2.0 mm in Ha or 5.3% in WR on SIT and SUP radiographs. The equivalence between these two cutoff values (2.0 mm in Ha vs. 5.3% in WR) slightly differ from our results. Niimi et al. [16] reported that a 2-mm change in vertebral height was the most reasonable cutoff value for screening for OVFs using a quantitative morphometric assessment of OVFs. In these two reports, the acute OVFs were primarily diagnosed by intensity changes on magnetic resonance images.

The four above-mentioned cutoff values for determination of bone union were briefly referred in chronological order, and the WR and WA values were converted into millimeters according to the equivalent values between Ha, WR, and WA obtained in the present study to compare these four cutoff values. Fujiwara et al. [17] defined that the cutoff value for determination of bone union was V-mobility of < 5% in WR on dynamic radiographs. It is likely that a cutoff value of < 5% in WR is equivalent to 1.5 mm in Ha according to the WR of 3.3% corresponding to 1.0 mm in Ha in this study. We defined the cutoff value for determination of bone union as V-mobility of ≤ 1.0 mm in Ha on dynamic radiographs [9, 18]. We thought that this cutoff value should be < 2.0 mm with reference to a cutoff value of 2.0 mm to diagnose acute OVFs reported by Kawasaki et al. [15]. A cutoff value of 1.0 mm in Ha was considered the nearest measurable value to bone union defined as OVFs without any visual change in the shape of the vertebral body as reported by Kishikawa [6] or as OVFs with no dynamic mobility in Ha as reported by Murata et al. [25]. Abe et al. [7] reported that 24 weeks after enrollment, vertebral instability within 5° in WA was defined as bone union because 5° was roughly detectable vertebral instability on dynamic X-ray examination performed by two observers in their preliminary study (*n* = 50), and the sensitivity and specificity for a cutoff value of 5° were 98% and 98%, respectively. The cutoff value of 5° in WA appeared to reflect V-mobility of just over 3 mm according to our results. Kitaguchi et al. [21] defined bone union as the absence of a vertebral cleft or abnormal motion (> 2.0 mm in Ha) 3 months after starting treatment.

These above-described Ha cutoff values for determination of bone union were < 1.0 mm [9, 18], < 1.5 mm [17], < 2.0 mm [21], and < 3.0 mm [7, 19, 20] when the WR (%) and WA (°) values were converted into millimeters. Because these cutoff values were different in each report, it will be difficult to determine which cutoff value is most appropriate for detection of bone union until such a cutoff value is established. It is expected that equivalent values between Ha, WR, and WA (i.e., 1.0 mm, 3.3%, and 1.5°, respectively) may be useful to establish cutoff values for determination of bone union hereafter as well as to secure a reliable value of V-mobility of OVFs by comparing and/or adjusting values measured simultaneously in three dimensions (Ha, WR, and WA) in clinical practice.

In our previous study, the WR and WA values equivalent to 1.0 mm in Ha were obtained on the assumption that the normal vertebrae and stable previous deformed OVFs were further deformed by 1.0 mm in Ha in the weight-bearing position. The WR values were calculated by the following formula: WR = Ha (1.0 mm)/Hp × 100 (%), in which 1.0 mm indicates the further deformed Ha value, and the WA values were calculated by trigonometry using Ha of 1.0 mm and the vertebral depth [23].

In the present study, however, the WR and WA values equivalent to 1.0 mm were obtained using V-mobility values of mobile non-united OVFs under treatment in actual clinical practice. Briefly, the WR and WA values equivalent to 1.0 mm in Ha were obtained by dividing the V-mobility values for WR and WA by the V-mobility for Ha, giving WR/HA and WA/Ha, respectively. Additionally, the WR/Ha value of 3.3% in the present study was very similar to that of 3.5%, and the WA/Ha value of 1.5° was the same as that in our previous study [23]. Therefore, it is very likely that the results obtained from the clinical OVFs in the present study have more clinical significance than those in the previous study and clarify the highly reliable equivalent values between Ha, WR, and WA.

These equivalent values can also be applied to those for V-deformity to determine the presence of incident vertebral fractures. Jalava et al. [26] defined an incident vertebral deformity as a vertebra with morphometric vertebral height of more than 3 SDs below the mean population norm for that vertebral level and a minimum decrease in height of 15% and 4.6 mm from the baseline. These decreased values of vertebral height presented as 15% in WR and 4.6 mm in Ha were found to be equivalent to our results; i.e., WR of 3.3% was equivalent to 1.0 mm in Ha.

### Study limitations

The mean values of the vertebral dimensions in grade 1 vertebrae were obtained in only seven vertebrae at L1 and L2 without any vertebrae at T11 and T12. Meanwhile, the WR and WA values equivalent to 1.0 mm in Ha in the present study were confirmed to be very similar to those in our previous study [23]. The mean WR/Ha value in the present study was 3.3%, which was slightly different from the WR/Ha of 3.5% in our previous study [23]. The difference between these two WR/Ha values was 0.2%; thus, WR/Ha of 3.5% or 3.3% and WA/Ha of 1.5° may be useful to obtain reliable values for V-mobility with reference to simultaneously measured dimensions (Ha, WA, and WR) in clinical practice, to compare the different reported cutoff values for evaluating V-mobility, and particularly to detect bone union. For standardization of OVF treatments, it is expected that the equivalent values between Ha, WR, and WA for V-mobility would be more precisely determined upon further measurements of larger numbers of OVFs, and that cutoff values to determine bone union would be established.

## Conclusion

The present results suggest that the mean WR and WA values equivalent to 1.0 mm in Ha are 3.3% and 1.5°, respectively, in grade 1–3 vertebrae in the T11–L2 region. These findings may be useful to secure a reliable value of V-mobility of OVFs using simultaneous measurements in three dimensions (Ha, WR, and WA) in clinical practice and to establish cutoff values for V-mobility to determine bone union.

## Data Availability

The datasets used and/or analyzed during the current study are available from the corresponding author on reasonable request.

## Abbreviations

DEC: Lateral decubitus position
Ha: Anterior vertebral height
Hp: Posterior vertebral height
OVF: Osteoporotic vertebral fracture
SIT: Sitting position
SUP: Supine position
V-deformity: Vertebral deformity
V-mobility: Vertebral mobility
WA: Wedge angle
WR: Wedge ratio

## References

[1] Horii C, Asai Y, Iidaka T, Muraki S, Oka H, Tsutsui S, Hashizume H, Yamada H, Yoshida M, Kawaguchi H, Nakamura K, Akune T, Tanaka S, Yoshimura N (2019). Differences in prevalence and associated factors between mild and sever vertebral fractures in Japanese men and women: the third survey of the ROAD study. J Bone Miner Metab.

[2] Hagino H (2021). Current and future burden of hip and vertebral fractures in Asia. Yonago Acta Med.

[3] Muratore M, Ferrera A, Masse A, Bistolfi A (2018). Osteoporotic vertebral fractures: predictive factors for conservative treatment failure. A systematic review. Eur Spine J.

[4] Longo UG, Loppini M, Denaro L, Maffulli N, Denaro V (2012). Conservative management of patients with an osteoporotic vertebral fracture: a review of the literature. J Bone Joint Surg Br.

[5] Ikumi A, Funayama T, Terajima S, Matsuura S, Yamaji A, Nogami U, Okuwaki S, Kawamura H, Yamazaki M (2021). Effects of conservative treatment of 2-week rigorous bed rest on muscle disuse atrophy in osteoporotic vertebral fracture patients. J Rural Med.

[6] Kishikawa Y (2012). Initial non-weight-bearing therapy is important for preventing vertebral body collapse in elderly patients with clinical vertebral fractures. Int J Gen Med.

[7] Abe T, Shibao Y, Takeuchi Y, Mataki Y, Amano K, Hioki S, Miura K, Noguchi H, Funayama T, Koda M, Yamazaki M (2018). Initial hospitalization with rigorous bed rest followed by bracing and rehabilitation as an option of conservative treatment for osteoporotic vertebral fractures in elderly patients: a pilot one arm safety and feasibility study. Arch Osteoporos.

[8] Sugita M, Takei H, Ishii J, Tsuchida H, Hiragami K, Takagi M (2019). Impact of strict rest management therapy with hospitalization on osteoporotic vertebral fractures. J Jpn Osteoporos Soc.

[9] Sato K, Yamada Y, Kogawa M, Sekiguchi T (2020). Vertebral mobility is a valuable indicator for predicting and determining bone union in osteoporotic vertebral fractures: a conventional observation study. J Orthop Surg Res.

[10] Newman M, Lowe CM, Barker K (2016). Spinal orthoses for vertebral osteoporosis and osteoporotic vertebral fracture: a systematic review. Arch Phys Med Rehabil.

[11] Kim HJ, Yi JM, Cho HG, Chang BS, Lee CK, Kim JH, Yeom JS (2014). Comparative study of the treatment outcomes of osteoporotic compression fractures without neurologic injury using a rigid brace, a soft brace, and no brace: a prospective randomized controlled non-inferiority trial. J Bone Joint Surg Am.

[12] Ebeling PR, Akesson K, Bauer DC, Buchbinder R, Eastell R, Fink HA, Giangregorio L, Guanabens N, Kado D, Kallmes D, Katzman W, Rodriguez A, Wermers R, Wilson HA, Bouxsein ML (2019). The efficacy and safety of vertebral augmentation: a second ASBMR Task Force Report. J Bone Miner Res.

[13] Kato T, Inose H, Ichimura S, Tokuhashi Y, Nakamura H (2019). Comparison of rigid and soft-brace treatments for acute osteoporotic vertebral compression fracture: a prospective, randomized, multicenter study. J Clin Med.

[14] McKiernan F, Jensen R, Faciszewski T (2003). The dynamic mobility of vertebral compression fractures. J Bone Miner Res.

[15] Kawasaki M, Tsuboya H, Kiyasu K, Ueta E, Takemasa R, Tani T (2008). Diagnostic accuracy of the plain radiography on sitting-supine position for fresh vertebral fracture. Kossetsu J Jpn Soc Fract Repair.

[16] Niimi R, Kono T, Nishihara A, Hasegawa M, Matsumine A, Kono T, Sudo A (2014). Efficacy of the dynamic radiographs for diagnosing acute osteoporotic vertebral fractures. Osteoporos Int.

[17] Fujiwara T, Kondo T, Nishimura M, Kurata T, Shiokawa Y (2011). Radiographic analysis of osteoporotic vertebral compression fractures: the fractures of delayed union. J Spine Res..

[18] Sato K, Yamashiro M, Kasama F, Matsuda M (2013). Conservative treatment of osteoporotic vertebral fractures: patient management in a recovery rehabilitation ward. Seikeigeka (Orthop Surg)..

[19] Nagashima K, Abe T, Shibao Y, Kumagai H, Miura K, Mataki K, Noguchi H, Funayama T, Koda M, Yamazaki M (2018). Clinical outcomes of conservative treatment for thoracolumbar vertebral fracture with glucocorticoid induced osteoporosis Comparison with primary osteoporosis patients. J Spine Res..

[20] Shibao Y, Abe T, Takeuchi Y, Nagashima K, Mataki K, Kumagai H, Miura K, Noguchi H, Funayama T, Amano K, Sakai S, Koda M, Yamazaki M (2019). Clinical outcomes of treatment for osteoporotic vertebral fracture with cleft. J Spine Res.

[21] Kitaguchi K, Kashii M, Ebina K, Sasaki S, Tsukamoto Y, Yoshikawa H, Murase T (2019). Effects of weekly teriparatide administration for vertebral stability and bony union in patients with acute osteoporotic vertebral fractures. Asian Spine J..

[22] Takahashi S, Hoshino M, Takayama K, Iseki K, Sasaoka R, Tsujio T, Yasuda H, Sasaki T, Kanematsu F, Kono H, Toyoda H, Nakamura H (2016). Predicting delayed union in osteoporotic vertebral fractures with consecutive magnetic resonance imaging in the acute phase: a multicenter cohort study. Osteoporos Int.

[23] Sato K, Kogawa M, Yamada Y, Yamashiro M, Kasama F, Matsuda M (2022). Equivalent values between anterior vertebral height, wedge ratio, and wedge angle in osteoporotic vertebral fractures. J Bone Miner Metab.

[24] Genant HK, Wu CY, van Kuijk C, Nevitt MC (1993). Vertebral fracture assessment using a semiquantitative technique. J Bone Miner Res.

[25] Murata K, Watanabe G, Kawaguchi S, Kanaya K, Horigome K, Yajima H, Morita T, Yamashita T (2012). Union rates and prognostic variables of osteoporotic vertebra fractures treated with a rigid external support. J Neurosurg Spine.

[26] Jalava T, Sarna S, Pylkkaenen L, Mawer B, Kanis JA, Selby P, Davies M, Adams J, Francis RM, Robinson J, McCloskey E (2003). Association between vertebral fracture and increased mortality in osteoporotic patients. J Bone Miner Res.

